# CAR T cell therapy for fighting IPF: perspectives on a living drug

**DOI:** 10.3389/fimmu.2026.1770081

**Published:** 2026-02-27

**Authors:** Wei Sun, Sirui Lu, Tao Chen, Yanrui He, Zuojun Xu, Zhigang Cai

**Affiliations:** 1 Department of Pulmonary and Critical Care Medicine, Second Hospital of Hebei Medical University, Shijiazhuang, Hebei, China; 2 Department of Respiratory and Critical Medicine, Peking Union Medical College Hospital, Chinese Academy of Medical Sciences and Peking Union Medical College, Beijing, China

**Keywords:** CAR T cell, fILD, ILD, IPF, therapy

## Abstract

Fibrotic interstitial lung disease (fILD), particularly idiopathic pulmonary fibrosis (IPF), represents an incurable progressive lung disorder characterized by a dismal prognosis. Fibroblasts constitute the principal cellular drivers of the fibrotic cascade. Although two pharmacological agents (pirfenidone and nintedanib) have secured regulatory approval for clinical application, they remain incapable of substantially attenuating disease progression. Persistent immune dysregulation driven alveolitis, occupies a critical upstream position in perpetuating fibroblast activation and extracellular matrix (ECM). Recent investigations have introduced an innovative strategy employing genetically engineered T cells to selectively target and eliminate activated fibroblasts. This approach involves generating chimeric antigen receptor (CAR) T cells *in vivo* by encapsulating mRNA encoding CARs within lipid nanoparticles (LNPs). These CAR T cells can specifically recognize and ablate fibroblasts expressing fibroblast activation protein (FAP). In this review, we summarize recently developed CAR T cell therapeutic strategies for IPF treatment with optimal targeting of FAP-fibroblasts, synthesize the existing preclinical studies and clinical trials evaluating anti-FAP CAR T cells to date, and critically discuss the adverse events associated with CAR T therapy alongside strategies to overcome current limitations of CAR T cell therapy in IPF management.

## 1 Introduction

The pathophysiological mechanisms underlying fibrotic interstitial lung disease (fILD) closely resemble those observed in idiopathic pulmonary fibrosis (IPF), suggesting the feasibility of shared therapeutic strategies (1). Pirfenidone and nintedanib exhibit both antifibrotic and anti-inflammatory properties, representing the first small-molecule drugs approved for IPF treatment (2). However, these agents do not provide curative benefits and are frequently associated with substantial tolerability concerns that limit their clinical utility (3).

Activated fibroblasts occupy a central position in the fibrogenic process, culminating in progressive accumulation of extracellular matrix (ECM) that compromises pulmonary structural integrity and functional capacity (4, 5). As the disease advances, immune system dysregulation ensues, whereby exaggerated immune responses promote the formation of activated fibroblast foci characteristic of fILD (6). Accumulating evidence demonstrates that newly recruited monocyte-derived macrophages substantially contribute to fILD pathogenesis and progression following alveolar epithelial cell (AEC) injury through the secretion of multiple profibrotic mediators, thereby rekindling interest in the pathogenic role of activated fibroblasts in IPF (7–9). These pathological fibroblasts are characterized by expression of fibroblast activation protein (FAP), which can be selectively targeted using radioactive tracer molecules (10). Preliminary investigations have demonstrated that tracers targeting (FAPI) exhibit remarkably high uptake rates in inflammatory responses and fibrotic lesions, establishing their diagnostic and potentially therapeutic utility (11, 12).

Targeted immunotherapy has recently emerged as a promising therapeutic option, particularly for patients demonstrating insufficient response to conventional treatments (13). Chimeric antigen receptor (CAR) T cell therapy represents a breakthrough innovation, delivering unprecedented outcomes in IPF management. The identification of FAP-positive cells as viable immunotherapeutic targets heralds a novel frontier in fILD treatment (14). Murine studies definitively confirm that CAR T cells can selectively ablate fibroblasts exhibiting elevated FAP expression, substantially reduce ECM deposition, and demonstrate minimal cytotoxicity toward normal tissue cells expressing low FAP levels, thereby effectively attenuating fILD progression (15). These compelling results provide a robust foundational rationale for the potential clinical application of FAP-targeted CAR T cells in IPF treatment.

This comprehensive review offers a systematic assessment of current and prospective applications of CAR T cell therapy in IPF, addresses existing and anticipated challenges confronting this therapeutic modality, and proposes a structured research framework to advance these promising therapeutic strategies toward clinical implementation.

## 2 A new cornerstone of CAR T cell therapy

**Figure 1:**
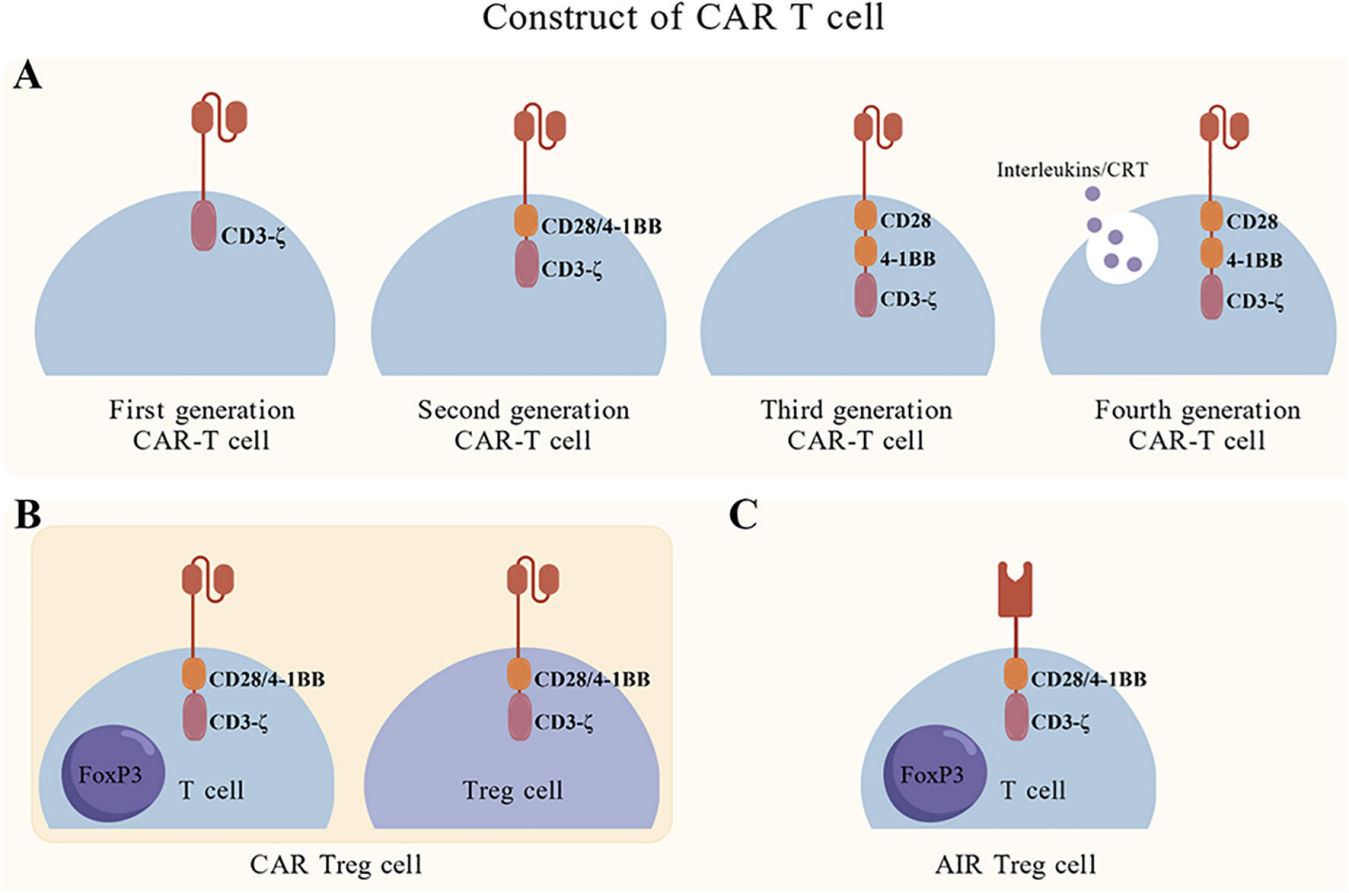
CAR T cell construct architectures. **(A)** Generational progression of CAR designs. Fourth-generation CARs integrate inducible cytokine production modules to enhance CAR T cell functionality and tumor microenvironment modulation. **(B)** Antigen-specific receptor construct variations: conventional CAR, chimeric autoantigen T cell receptor (CATCR), and split universal programmable (SUPRA) CAR T cells. **(C)** Constructs of CAR regulatory T cells (CAR-Tregs) and artificial immune receptor (AIR)-equipped Tregs for immunomodulation. AIR, artificial immune receptor; CAAR, chimeric autoantibody receptor; CAR, chimeric antigen receptor; CATCR, chimeric autoantigen T cell receptor; SUPRA, split universal programmable; Treg, regulatory T cell.

CAR T cell therapy harnesses the synergistic power of T cell-mediated cytotoxicity combined with antibody-derived specificity to precisely target and eradicate diseased cells (16). The single-chain variable fragment () confers antigen specificity, whereas intracellular signaling domains orchestrate T cell-mediated cytotoxic responses (17). Advances in synthetic biology and protein engineering have progressively refined CAR architectural designs for tailored clinical applications (**Figure 1**). Initial first-generation CARs incorporated a CD4 extracellular domain fused to the CD3ζ signaling domain; however, these constructs demonstrated limited therapeutic efficacy and inadequate T cell persistence (18). Subsequently, second-generation CARs integrated co-stimulatory domains, such as CD28 or 4-1BB, substantially enhancing effector functions, cytokine production, and long-term persistence (19). Consequently, several second-generation CAR T cell products have received US FDA approval for treating various hematological malignancies (20). Further receptor modifications-including removal of inhibitory checkpoint domains, incorporation of dominant-negative receptors, or strategic mutation of co-stimulatory domains-have been systematically devised to optimize therapeutic potency while minimizing toxicity (21).

The remarkable success of CAR T cells in refractory clinical scenarios underscores the emergence of cell and gene therapies as foundational pillars in modern oncology (22). CAR T cells exhibit distinctive attributes that fundamentally distinguish them from traditional pharmacological therapies (22). Their intrinsic target specificity substantially surpasses that of current small-molecule drugs, enabling precise recognition of disease-associated antigens (23). CAR T cells leverage the inherent cytotoxic machinery of T lymphocytes, exploiting highly efficient endogenous immunological mechanisms for target cell elimination (24). A particularly remarkable advantage of these “living therapies” resides in their capacity for exponential clonal expansion: a single CAR T cell can potentially eliminate hundreds to thousands of target cells through serial cytotoxic encounters (25). Moreover, the CAR T cell population physiologically contracts following antigen clearance in the absence of persistent antigenic stimulation, yet retains the capacity to maintain long-term immunosurveillance spanning multiple years through memory T cell formation (26). Clinical feasibility and safety are substantiated by data accumulated from over 15, 000 treated patients worldwide, without documented occurrences of autologous CAR T cell malignant transformation (26, 27). This remarkably favorable safety profile proves especially encouraging given the typically high disease burden in oncology applications, suggesting broader therapeutic applicability for diseases characterized by lower target cell loads (28). Thus, the exceptional specificity, potent cytotoxicity, self-amplification capacity, and established clinical safety profile of CAR T cells present compelling opportunities for therapeutic application across diverse pathological conditions beyond oncology (28).

## 3 Target selection for CAR T cell therapy in IPF

### 3.1 Immune dysregulation sustaining fibroblast activation in IPF

Considering that chronic epithelial injury and dysfunctional wound healing collectively trigger the fibrotic cascade, fibroblasts-one of the principal non-immune cell populations implicated in tissue repair-occupy a pivotal position in fILD pathogenesis (29). Based on single-cell RNA sequencing (scRNA-seq) analysis and DNA methylation profiling of fibroblast phenotypes, activated fibroblasts are increasingly recognized as heterogeneous cellular populations endowed with profibrotic properties (30, 31). The unrelenting activation of fibroblasts in fILD encompasses multiple pathological processes, including fibroblast-to-myofibroblast transformation (FMT), enhanced migratory capacity, resistance to apoptotic clearance mechanisms, and excessive deposition of ECM proteins that distort normal lung architecture (32, 33).

Numerous metabolites and soluble paracrine factors produced by macrophages are conventionally regarded as essential mediators orchestrating the biological transition between macrophage polarization states and fibroblast activation-collectively termed macrophage-fibroblast crosstalk-during the pathological progression of fILD (34). S100a4, also designated fibroblast-specific protein-1 (FSP-1), was initially considered a protein exclusively expressed by fibroblasts; however, contemporary experimental evidence indicates that it can potently induce fibrogenic phenotypes in mesenchymal progenitor cells in IPF (35–37). Recent findings suggest that S100a4 released from macrophages contributes substantially to fILD pathogenesis by promoting fibroblast differentiation and activation (35). Furthermore, the abundant expression of CX3CR1 in macrophage populations has prompted researchers to systematically evaluate the functional significance of the CX3CL1–CX3CR1 chemokine axis in fILD (38, 39). Although CX3CR1 deficiency may not quantitatively impact macrophage populations following bleomycin (BLM) administration, it nevertheless profoundly influences macrophage polarization and subsequent fibrotic processes, including myofibroblast activation and ECM remodeling (40).

**Figure 2:**
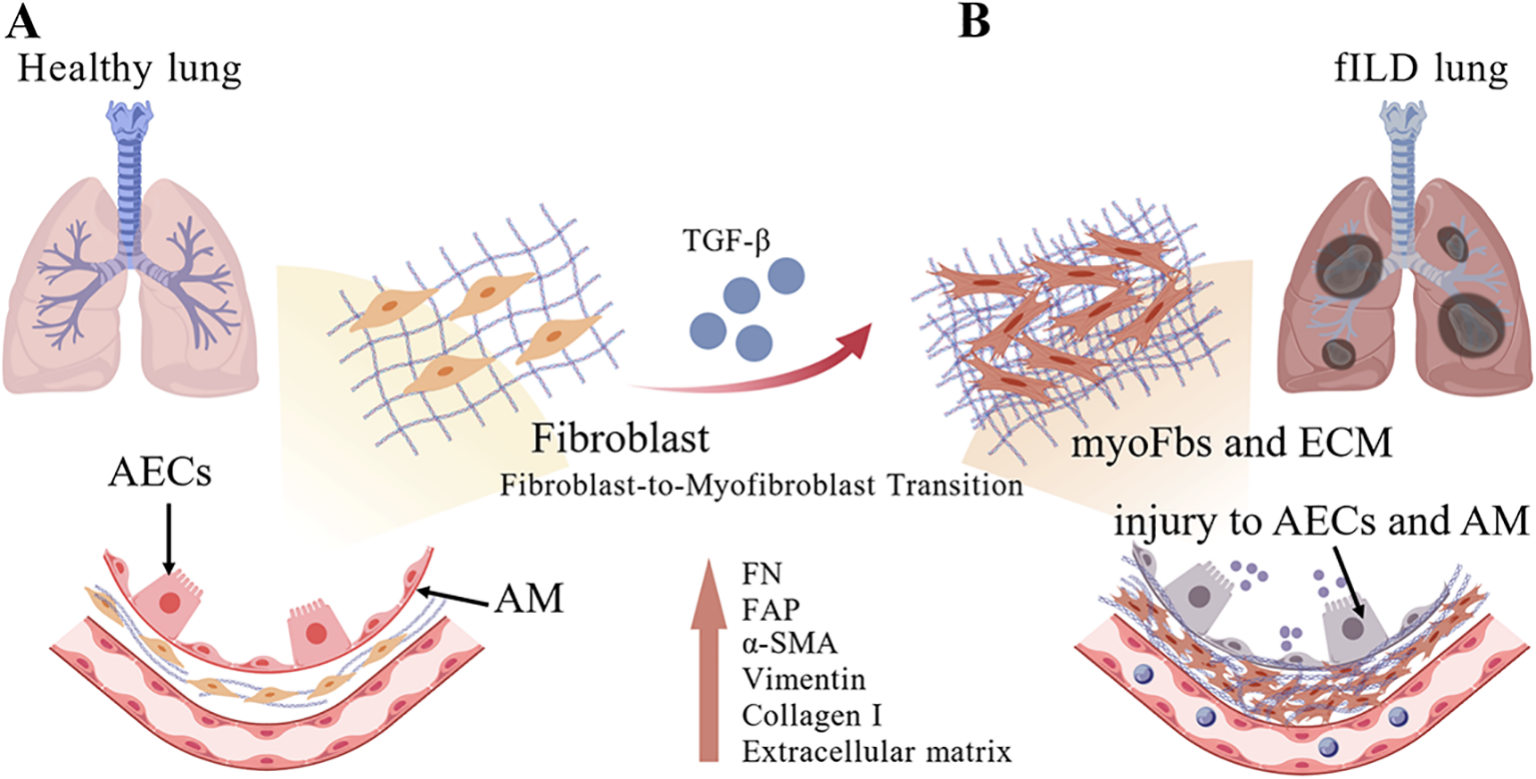
The pulmonary microenvironment in IPF exhibits increased pro-fibrotic mediators that induce fibroblast transformation into myofibroblasts **(myoFbs)** and progressive ECM deposition. **(A)** Physiological crosstalk among alveolar AECs, AM, and resident fibroblasts maintains normal lung architecture and ECM homeostasis. **(B)** Persistent injury to AECs and AM precipitates the secretion of fibrotic cytokines, predominantly TGF-β1, driving sustained upregulation of FAP in FAP-labeled fibroblasts alongside enhanced expression of myoFb markers, including α-smooth muscle actin (α-SMA) and collagen type I, indicative of pathological fibroblast-to-myoFb transition characteristic of fibrotic lung tissue. AECs, alveolar epithelial cells; AM, Alveolar Macrophages; ECM, extracellular matrix; myoFbs, myofibroblasts; TGF-β, transforming growth factor-β; α-SMA, α-smooth muscle actin.

Under certain pathological conditions, neither pro-inflammatory cytokine deficiency nor aggressive steroid and immunosuppressive therapeutic regimens can effectively limit fILD progression (41, 42). Based on accumulating research evidence, it has become imperative to consider that the establishment of an immunoregulatory microenvironment-predominantly comprising regulatory lymphocytes and myeloid-derived suppressor cells-may be fundamentally associated with pathological pulmonary fibrosis (43, 44). Within the type 2 immune response framework, interleukin-10 (IL-10) functions as an inducer of fibrogenic cellular phenotypes, particularly promoting M2-like and Th2-like pro-fibrotic cell differentiation (45, 46). Consistent with the actions of transforming growth factor-β1 (TGF-β1), the predominant profibrogenic cytokine, investigations have revealed that the anti-inflammatory cytokine IL-10 is abundantly produced by alveolar macrophages (46, 47). Additional studies have further demonstrated that IL-10 paradoxically suppresses lung fibrosis through TGF-β1-dependent mechanisms, wherein IL-10 overexpression simultaneously regulates macrophages polarization via the CCL2/CCR2 chemokine axis (48). In this context, accumulating evidence suggests that immunoregulatory cytokines-paradigmatically TGF-β1 and IL-10-serve as principal orchestrators of the pro-fibrotic microenvironment, triggering cascades of fibroproliferative wound healing processes intimately linked to inappropriate communication between M2-polarized macrophages and activated fibroblasts within pulmonary tissues (**Figure 2**) (49, 50). Phenotypic distinctions between macrophage subpopulations in normal versus fibrotic lungs substantiate the concept of specific innate immune factors playing causal roles in fILD pathogenesis (51, 52). To emphasize this critical point, co-localization analyses and causal modeling approaches have been systematically employed to identify macrophages highly expressing secreted phosphoprotein 1 (SPP1) and MER proto-oncogene tyrosine kinase (MERTK)—designated SPP1hi macrophages—and these identified macrophage subsets prove crucial in accelerating fibroblast activation within lung tissues (51, 53).

Considering that intercellular communication mediated through metabolite exchange has been extensively characterized in cancer cells and carcinoma-associated fibroblasts, it becomes necessary to provide mechanistic insights into the role of immunometabolic modulators in fILD as intercellular messengers governing macrophage-fibroblast interactions (51, 54). Glycolytic upregulation in lung fibroblasts, smooth muscle cells, and endothelial cells has been causally implicated in the pathological progression of pulmonary fibrosis (55). Subsequent metabolomic analyses have demonstrated significant elevation of glycolysis-derived lactate in conditioned media from TGF-β1-induced lung myofibroblasts and in bronchoalveolar lavage fluid (BALF) from murine fibrosis models (56–58). Macrophages cultured with fibroblast-conditioned media undergo phenotypic switching toward pro-fibrotic states characterized by elevated expression of select pro-fibrotic mediators, thereby establishing a positive feedback loop wherein myofibroblast glycolysis accelerates the pro-fibrotic activity of macrophages in fILD (59, 60). Fatty acid (FA) metabolism in cells regulates diverse biological activities and energy production through FA oxidation pathways (61). Altered FA metabolite concentrations and compositional profiles have been consistently identified in patients with fILD and in experimental animal models, suggesting the critical role of lipid metabolism in promoting pro-fibrotic phenotypes in both macrophages and fibroblasts/myofibroblasts (62, 63). For example, macrophage polarization toward the M2 phenotype depends not only upon metabolic energy supplied through FA oxidation but is also transcriptionally activated by peroxisome proliferator-activated receptor gamma (PPAR-γ), a transcription factor initially identified in adipose tissue for its regulatory role in FA storage and lipid homeostasis (64, 65). Additionally, genetic cell lineage tracing studies targeting lung fibroblasts in mice have revealed that both abundant pro-fibrotic cytokines secreted by polarized macrophages and PPAR-γ activation can induce phenotypic switching between lipid-laden fibroblasts and contractile myofibroblasts during both progression and resolution phases of lung fibrosis (66, 67).

### 3.2 Targeting FAP-expressing fibroblasts as the primary focus of CAR T cell therapy

Previous investigations have conclusively demonstrated that FAP is aberrantly expressed in lesional tissues of fILD, with particularly prominent expression within fibrotic interstitium and concentrated in fibroblast foci characteristic of IPF patients (68). FAP belongs to the prolyl peptidase enzyme family, sharing structural and functional homology with dipeptidyl peptidase IV (DPPIV), with which it exhibits approximately 70% amino acid sequence identity (69). Regarding its endopeptidase enzymatic activity, FAP paradoxically possesses antifibrotic proteolytic capacity, as it functionally complements matrix metalloproteinase 1 (MMP1) to proteolytically cleave fibrillar collagens type I and III. Moreover, a soluble circulating form of FAP has been identified and designated anti-plasmin cleaving enzyme (APCE) (70). APCE functions analogously to membrane-bound FAP and synergistically complements MMP1 in collagen degradation. Additionally, APCE proteolytically cleaves the N-terminal region of α2-antiplasmin, generating a modified substrate that enhances clot stability by reducing blood clot degradation and delaying fibrinolytic processes. Notably, human and murine FAP share approximately 89% amino acid sequence homology and exhibit comparable enzymatic activities, facilitating translational research between species (71).

The chronic bleomycin-induced fibrosis model possesses the distinct advantage of inducing progressive, irreversible lung fibrosis with histopathological lesions that more closely recapitulate those observed in IPF patients compared to acute injury models (72). Investigations employing both FAP-overexpressing and FAP knockout (FAPKO) transgenic mouse lines revealed exacerbated fILD severity, with FAPKO mice exhibiting enhanced pulmonary infiltration of immune cell populations (73). However, these studies did not observe statistically significant differences in fibrosis severity between transgenic and wild-type (WT) mice when fILD was induced through constitutive expression of active TGF-β (74). This suggests that bleomycin preferentially activates fibroblasts into FAP-positive cells exhibiting a proteolytic phenotypic profile, whereas TGF-β signaling predominantly mediates fibroblast-to-myofibroblast differentiation and subsequent tissue contractility (74). Conversely, Egger and colleagues observed pro-fibrotic functional properties attributable to FAP (12). They induced fibrosis in WT mice using the chronic bleomycin administration model and therapeutically treated animals with PT100, a pharmacological FAP inhibitor. PT100-treated mice demonstrated significantly improved survival and exhibited quantitatively reduced pulmonary fibrotic areas compared to vehicle-treated controls (12). It merits noting that PT100 functions as a pan-inhibitor targeting the entire dipeptidyl peptidase (DPP) enzyme family rather than exclusively inhibiting FAP. The contradictory experimental outcomes may therefore be mechanistically explained by this lack of pharmacological specificity, which contrasts with the genetic precision afforded by mouse models employing FAPKO mice or selective depletion strategies targeting FAP-positive cells (75).

**Figure 3:**
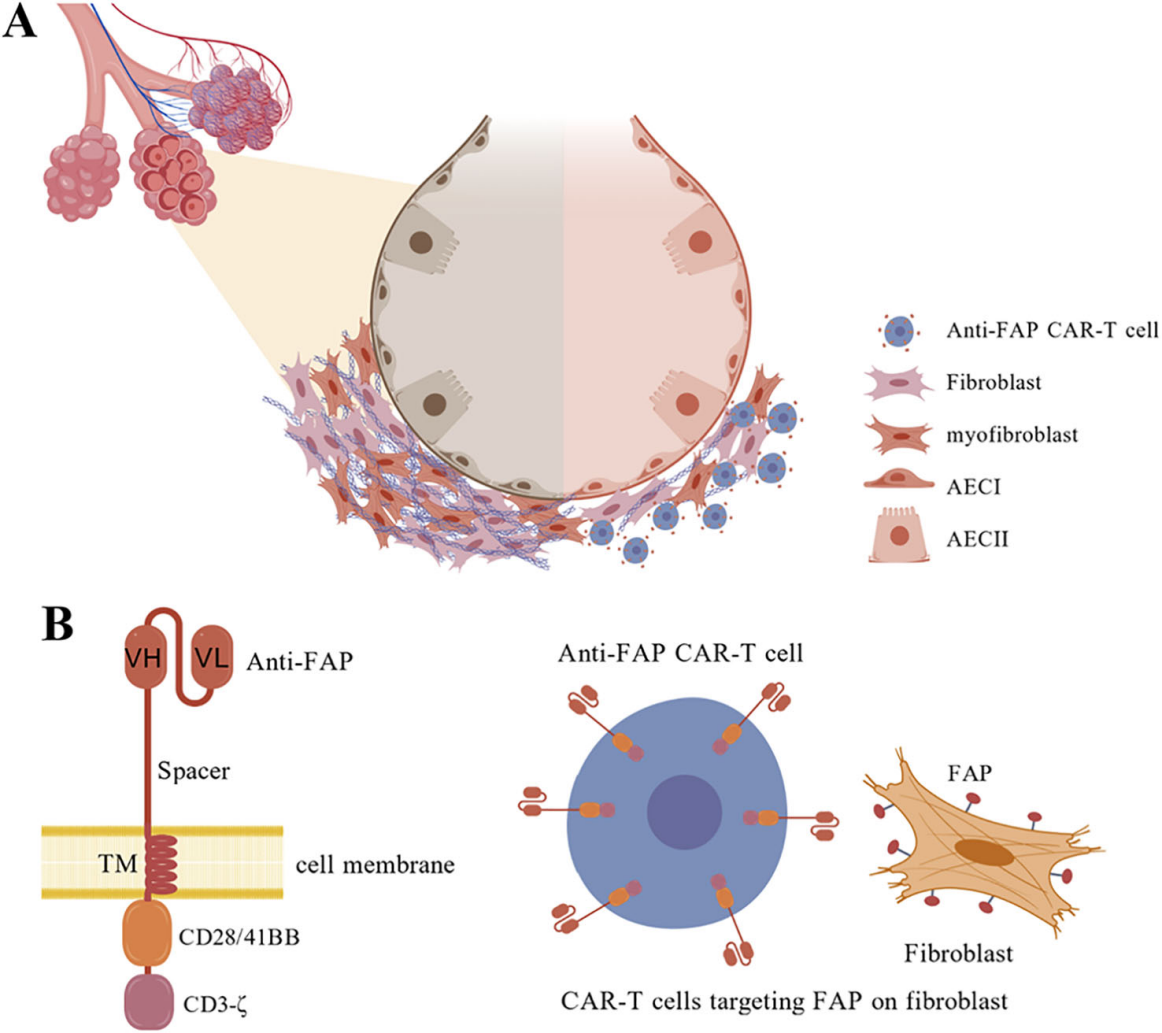
Anti-FAP CAR T cells targeting myofibroblasts (myoFbs) in pulmonary fibrosis. **(A)** Schematic representation of anti-FAP CAR T cell engagement with FAP antigen expressed on myoFbs within the fibrotic pulmonary microenvironment, leading to targeted cytolytic activity. **(B)** Molecular architecture of anti-FAP CAR construct composed of an anti-FAP single-chain variable fragment (scFv) conferring antigen specificity, extracellular spacer domain, transmembrane domain (TM), and intracellular signaling domains (CD28 or 4-1BB co-stimulation coupled with CD3ζ activation). The engineered CAR construct is transduced via viral vectors or mRNA delivery and stably expressed on T cell surfaces. Generated anti-FAP CAR T cells bind with high specificity to FAP antigen expressed on activated myoFbs, triggering targeted cytotoxic elimination.

FAP serves as a cell surface antigen marker denoting fibroblast activation state in cardiac muscle tissue and in pulmonary fibrotic lesions (75). It can be strategically exploited as a target molecule for engineering CAR T cells designed for IPF therapeutic intervention (**Figure 3**). A pioneering research team has successfully developed a CD5-targeted LNP-encapsulated FAP CAR mRNA delivery system (76). Given that CD5 is physiologically expressed on cell membranes of T lymphocytes and a minor subset of B lymphocytes, and considering it is dispensable for T lymphocyte effector functions, investigators conjugated CD5-specific antibody molecules to the LNP surface, thereby enabling the CD5-specific antibody-modified LNP-mRNA delivery platform to selectively target CD5-positive T lymphocytes *in vivo* (77). Further mechanistic investigations revealed spatial clustering of FAP CAR-positive T lymphocytes with FAP-positive activated fibroblasts within fibrotic lesions, accompanied by substantially reduced fibrosis severity in regions surrounding these cellular clusters, whereas fibrotic tissue architecture in spatially distant areas remained unaltered (78). This landmark study validated both the functional efficacy and T lymphocyte-targeting capability of the LNP-mRNA delivery system at the cellular and tissue levels, and for the first time experimentally demonstrated the feasibility of generating functional CAR T cells *in vivo* without ex vivo manipulation.

It warrants particular emphasis that CAR expression mediated by the CD5-targeted LNP-FAP CAR mRNA delivery system exhibits inherently transient kinetics, resulting in temporally limited CAR T cell persistence *in vivo* (77, 79). Should enhanced or sustained therapeutic efficacy be clinically required, repeated administrations can be safely performed to maintain therapeutic CAR T cell levels (77). For CAR T cell therapeutic applications in non-malignant diseases such as IPF, autoimmune disorders, and infectious diseases, transient CAR T cell expression profiles prove advantageous by substantially mitigating risks of long-term toxicities and enabling superior therapeutic controllability (80, 81). During clinical implementation, this transient expression strategy may afford greater dose titration flexibility and improved safety-efficacy balance (82). Consequently, transiently expressed CAR T cell platforms possess considerable developmental potential for future clinical translation in non-oncological indications.

## 4 *In vivo* application of CAR T cell therapy for IPF treatment

IPF represents a devastating progressive lung disease with alarmingly high global incidence and mortality rates (83). Its pathological hallmark involves hyperactivated fibroblasts driving aberrant accumulation of fibrotic tissue, causing progressive deterioration of lung architecture and functional capacity, ultimately culminating in respiratory failure and premature death (83). Currently, clinical therapeutic options available for IPF management remain exceedingly limited and inadequate. Conventional pharmacological agents can merely provide symptomatic palliation but fundamentally cannot reverse the underlying fibrotic processes or substantially alter the disease trajectory (84).

Jing Yan and colleagues successfully generated transient FAP CAR T cells *in vivo* utilizing the LNP-mRNA delivery platform (85). Through CD5-targeted modification enabling specific T cell transfection within splenic compartments, they achieved selective elimination of activated fibroblasts, restoration of physiological mechanical properties of the ECM, and promotion of alveolar epithelial cell polarization alongside beneficial immune microenvironment remodeling (15). Compared with the conventional antifibrotic drug pirfenidone, this innovative CAR T cell-based therapy demonstrated substantially superior efficacy in reversing established IPF (15). Initially, the research team systematically optimized LNP compositional formulations. By replacing cholesterol with equimolar quantities of β-sitosterol-a plant-derived sterol, they successfully enhanced mRNA expression efficiency and cellular transfection rates (15). Experimental optimization revealed that when β-sitosterol constituted 30% of total lipid composition, LNPs achieved maximal mRNA expression levels across diverse mammalian cell lines tested. Building upon this optimized formulation, investigators further functionalized the LNP surface through covalent conjugation of CD5-specific monoclonal antibodies, thereby conferring selective T cell-targeting capability. Subsequently, the research team rationally designed two distinct FAP-targeting CAR molecular architectures: an optimized transmembrane domain construct (FAP CAR 2.2) incorporating 4-1BB co-stimulation and an alternative CD28-based co-stimulatory domain construct (FAP CAR 2.1). Following 24-hour co-incubation of CD5-targeted β-sitosterol-containing LNPs (CD5/βLNP) with isolated murine T cells, approximately 80% of T cells successfully expressed functional FAP CAR receptors on their plasma membranes. These engineered FAP CAR T cells demonstrated specific cytolytic activity against NIH 3T3 fibroblast cells engineered to express FAP, with cytotoxicity exhibiting dependence on effector-to-target (E: T) cell ratios. Maximal cytolytic efficiency was achieved at an E: T ratio of 10:1, demonstrating potent and specific anti-fibroblast activity (15). Comparative functional analyses of different CAR domain architectures revealed that T cells expressing FAP CAR 2.2 released significantly reduced quantities of inflammatory cytokines compared to FAP CAR 2.1-expressing counterparts, while maintaining equivalent cytotoxic capacity against target cells. This advantageous safety profile positions FAP CAR 2.2 as the superior construct for clinical translation. Additionally, kinetic analyses demonstrated that FAP CAR mRNA delivered via CD5/βLNP achieved peak expression levels in T cells within 24 hours post-administration and was substantially cleared by 96 hours, confirming the transient expression characteristics desirable for non-oncological applications (15). Collectively, these results establish that FAP CAR 2.2 provides an optimized safety and efficacy profile for *in vitro* functional applications targeting IPF treatment.

In comprehensive animal model experimental studies, researchers established fILD models in both young adult (8-week-old) and aged (16-month-old) mice to recapitulate human age-related disease heterogeneity, subsequently administering CD5/βLNP-FAP CAR 2.2 therapeutically to diseased animals. Experimental results convincingly demonstrated that compared with pirfenidone—the current standard-of-care antifibrotic pharmaceutical agent—CD5/βLNP-FAP CAR 2.2 treatment achieved significantly more pronounced reversal of established fibrosis within abbreviated timeframes, substantially increased animal survival rates, improved body weight maintenance, and reduced lung coefficient measurements indicative of reduced fibrotic burden. Therapeutic efficacy proved consistently robust across both young and aged mouse model cohorts, demonstrating broad applicability across age spectrums. Following CD5/βLNP-FAP CAR 2.2 therapeutic intervention, hydroxyproline content, a biochemical marker of collagen deposition-in pulmonary tissues, approximated levels observed in healthy control animals. Concurrently, biomechanical analyses revealed significant reductions in elastic modulus measurements of lung tissue, and ECM stiffness was markedly diminished, collectively indicating effective improvement of pathological tissue mechanical properties. Furthermore, protein expression analyses demonstrated substantial downregulation of fibrosis-associated and pro-inflammatory protein markers, whereas anti-inflammatory and tissue-reparative protein expression was correspondingly upregulated, indicating successful suppression of the pathological inflammatory microenvironment and promotion of physiological ECM homeostatic recovery processes. Additionally, flow cytometric and immunohistochemical analyses revealed increased populations of apolipoprotein E-positive (Apoe+) macrophages within lung tissues following CD5/βLNP-FAP CAR 2.2 treatment. These Apoe+ macrophages, signaling through the Apoe-TREM2 (triggering receptor expressed on myeloid cells 2) axis, facilitate differentiation of monocyte-derived macrophages and maintain phenotypic stability of tissue-resident alveolar macrophages, thereby contributing to the resolution of pathological inflammation (15). Concomitantly, pulmonary tissue demonstrated increased infiltration of effector T cell populations, including T helper 1 (Th1) and cytotoxic T (Tc) cells, which exert important immunomodulatory and tissue-reparative functions beneficial for IPF therapeutic outcomes.

In clinical oncology practice, CAR T cell therapy has achieved transformative success in treating hematological malignancies and certain cancers (86). However, its therapeutic application in solid organ fibrotic diseases such as pulmonary fibrosis remains in early exploratory phases and confronts substantial translational limitations. These constraints not only complicate the interpretation of current preclinical CAR T cell therapy data but also severely restrict clinical scalability and broader applicability (87). These fundamental limitations compel critical re-examination and potential revision of CAR T cell therapeutic paradigms derived from murine experimental results. Firstly, in the context of pulmonary fibrosis, the therapeutic objective of CAR T cell intervention may not necessitate complete ablation of all target cell populations; rather, the goal should emphasize precise immunomodulation or beneficial resetting of the pathological tissue microenvironment. For instance, engineering CAR T cells capable of secreting antifibrotic growth factors (such as hepatocyte growth factor, HGF) or expressing matrix-degrading enzymes, or implementing controllable “switch” systems enabling temporal regulation of fibroblast-targeting activity, may prove more physiologically appropriate. Secondly, the stringent “complete remission” criteria routinely applied in oncology-derived murine experiments do not translate appropriately to pulmonary fibrosis therapeutic contexts. Clinically relevant therapeutic endpoints should instead prioritize stabilization or measurable improvement of pulmonary function parameters (such as forced vital capacity, FVC), patient-reported quality of life metrics, and quantifiable reduction of fibrotic lesion burden on high-resolution computed tomography imaging. Moreover, extended longitudinal observation periods will be essential to comprehensively assess long-term therapeutic durability and safety. Thirdly, given that IPF patients already suffer from severely compromised respiratory reserve, any potential severe treatment-related toxicity could prove clinically unacceptable or even life-threatening. This safety imperative necessitates that preclinical animal models demonstrate substantially improved capacity to accurately predict pulmonary immune-mediated toxicities in human patients. Finally, monotherapy approaches utilizing CAR T cells alone will likely prove insufficient for achieving durable therapeutic responses in complex fibrotic diseases. Future therapeutic paradigms must embrace rational combination treatment strategies, such as integrating CAR T cell therapy with established antifibrotic pharmacological agents (nintedanib, pirfenidone), anti-inflammatory immunomodulators, or ECM-targeting biologics specifically designed to enhance CAR T cell tissue infiltration and persistence within fibrotic microenvironments.

In summary, through the optimized CD5/βLNP-FAP CAR 2.2 platform, transient *in vivo* generation of functional FAP CAR T cells has been successfully achieved, enabling effective elimination of FAP-overexpressing activated fibroblasts, substantial amelioration of pulmonary fibrosis severity, and promotion of beneficial lung tissue regeneration alongside immune functional reconstruction. This significant scientific achievement not only demonstrated robust therapeutic efficacy across multiple preclinical animal model systems but also establishes a compelling scientific foundation supporting future clinical translation for IPF patient treatment. This innovative therapeutic approach holds considerable promise for bringing transformative hope to patients suffering from progressive pulmonary fibrosis and related fibrosing interstitial lung diseases.

## 5 Monitoring and follow-up considerations in CAR T cell therapy

### 5.1 Current challenges confronting CAR T cell therapy for IPF

Current preclinical animal models inadequately recapitulate the pathological complexity and pronounced heterogeneity characteristic of human fILD (88). The pulmonary microenvironment in fILD comprises extraordinarily diverse cellular populations-including phenotypically distinct fibroblast subsets, myoFbs, microvascular and lymphatic endothelial cells, and heterogeneous immune cell infiltrates-alongside biochemically varied ECM components exhibiting spatial and temporal heterogeneity (89–91). Substantial inter-patient and intra-patient heterogeneity in the pulmonary fibrotic microenvironment across fILD subtypes and progressive disease stages considerably complicates the rational design of broadly effective targeted immunotherapies (92). Unlike oncology applications, where abundant tumor-specific or tumor-associated antigens facilitate highly selective targeted treatments, fILD pathogenesis exhibits a relative paucity of validated disease-specific antigenic targets, thereby substantially restricting the therapeutic applicability of engineered immune cell platforms (including CAR T cells, CAR-equipped macrophages, or engineered natural killer cells) for clinical translation in fibrotic diseases (93–95). Additionally, immune cell-based therapeutic interventions carry inherent risks of precipitating immune-related adverse events, including cytokine release syndrome (CRS), immune effector cell-associated neurotoxicity syndrome (ICANS), and graft-versus-host disease (GVHD), with risks particularly elevated when employing allogeneic cell products (96). Consequently, stringent safety considerations assume paramount importance in fILD therapeutic applications to avoid inadvertently exacerbating pre-existing organ damage and to rigorously minimize off-target effects on healthy tissues.

Autologous CAR T cell manufacturing represents an emerging yet prohibitively expensive biotechnology platform (97). The substantial financial expenditures associated with cell product engineering, large-scale manufacturing under current Good Manufacturing Practice (cGMP) conditions, quality control testing, and clinical administration frequently exceed several hundred thousand US dollars per patient, presenting formidable economic barriers even for potentially curative therapeutic interventions (97). These extraordinarily high costs correlate directly with severely limited patient accessibility, effectively restricting CAR T cell therapy availability to a select number of specialized academic medical centers and comprehensive cancer centers possessing the requisite infrastructure and expertise (98). This personalized “living drug” manufacturing paradigm necessitates highly specialized cell processing facilities, sophisticated quality assurance systems, and multidisciplinary clinical expertise, thereby confining practical availability to a geographically limited number of major tertiary care centers (98). Consequently, substantial patient populations who might represent ideal candidates for CAR T cell therapy based on disease characteristics currently cannot access this potentially transformative treatment modality due to geographic, financial, or healthcare system-related barriers.

### 5.2 Safety precautions and toxicity management for CAR T cell therapy

CRS constitutes the most clinically significant and frequently encountered toxicity syndrome associated with CAR T cell therapy (99). Before clinical application in novel disease contexts beyond oncology, rigorous preclinical and early-phase clinical evaluation of CRS-like symptomatology is essential (100, 101). Early-phase CAR T cell clinical trials conducted in systemic lupus erythematosus (SLE) patients reveal that comparatively lower target cell burdens characteristic of non-malignant autoimmune indications may potentially result in attenuated CRS severity compared to oncology applications; however, larger randomized controlled trials with extended follow-up are required for definitive confirmation of this hypothesis (102). Preclinical CAR T cell studies specifically targeting activated cardiac fibroblasts in heart failure animal models demonstrated the absence of serum cytokine elevations, no alterations in body weight parameters, and no impairment of physiological wound healing processes in treated murine subjects (103). Nevertheless, in human patients, therapeutic reduction of substantial established fibrotic tissue burdens could conceivably provoke clinically significant CRS-like inflammatory responses, warranting careful monitoring and potential intervention.

Therapeutic depletion of healthy cell subsets-exemplified by B cell ablation in SLE patients treated with anti-CD19 CAR T cells-may paradoxically confer clinical benefit through elimination of autoreactive B cell clones; however, long-term immunological consequences and potential infectious complications remain incompletely characterized (102). Preliminary clinical data emerging from ongoing trials suggest these cellular depletions may prove transient with spontaneous immune reconstitution; however, long-term follow-up studies spanning multiple years will be necessary to definitively clarify potential chronic complications, including hypogammaglobulinemia and opportunistic infections (81). Although pathological chronic fibrosis unequivocally drives organ dysfunction, it remains uncertain whether specific physiological contexts exist wherein fibroblast activation serves beneficial homeostatic functions that should be preserved (103). Senescent cells, while contributing to age-related pathologies, nevertheless perform essential physiological roles during embryonic development, acute wound healing responses, and tissue regeneration processes (104). Therefore, comprehensive evaluation of potential impacts of senolytic CAR T cell therapies on these beneficial physiological processes constitutes a critical research priority, emphasizing the importance of selectively targeting pathological senescent cell populations through identification and validation of disease-specific antigens (104). Transient CAR T cell expression platforms prove particularly attractive in non-oncological contexts, as inherently limited CAR T cell persistence substantially alleviates concerns regarding potential long-term safety risks associated with persistent CAR expression and chronic immune activation (105).

**Figure 4:**
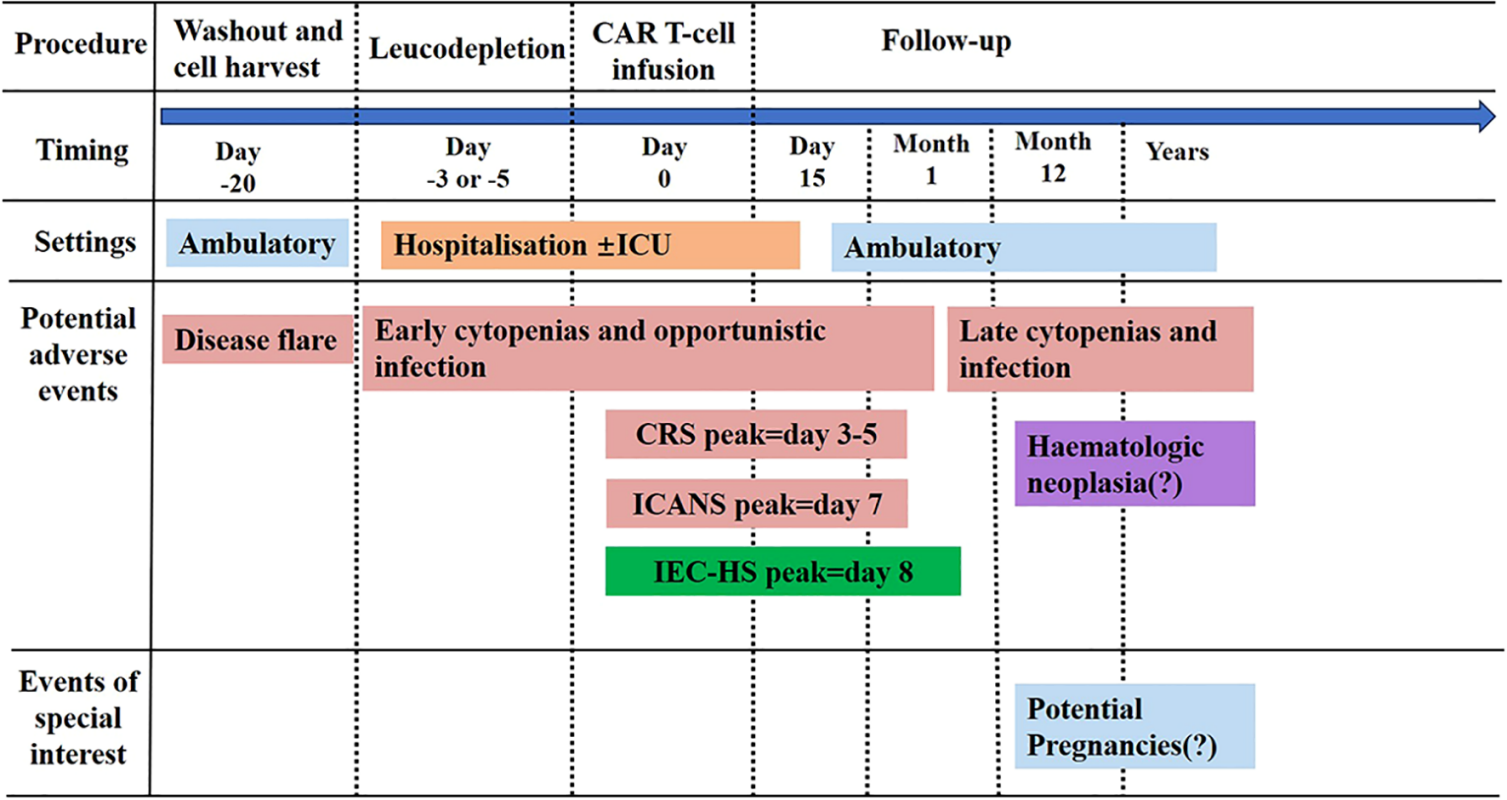
Comprehensive safety considerations in CAR T cell therapies for non-oncological applications. IEC-HS can manifest between day 4 and day 32 post-infusion. CAR, chimeric antigen receptor; CRS, cytokine release syndrome; ICANS, immune effector cell-associated neurotoxicity syndrome; ICU, intensive care unit; IEC-HS, immune effector cell-associated hemophagocytic lymphohistiocytosis-like syndrome.

The rapidly expanding field of CAR T cell therapy beyond oncology encompasses numerous fundamental unanswered questions demanding systematic investigation in the forthcoming years (106). Determination of the optimal therapeutic index remains a pivotal research focus, given the criticality of appropriate CAR T cell infusion dosing strategies to achieve an optimal balance between therapeutic efficacy and acceptable safety profiles, particularly for mRNA-based transient CAR T cell platforms requiring repeated administrations (107). In both acute injury-driven and fILD, the clinical application paradigm for transient CAR T cell immunotherapy necessitates careful evaluation through well-designed dose-finding and pharmacodynamic studies. Optimal therapeutic timing relative to disease stage and progression kinetics remains uncertain and likely varies substantially according to underlying disease pathogenesis and individual patient factors (108). It proves imperative to definitively establish whether irreversible advanced disease stages exist beyond which therapeutic intervention becomes ineffective or potentially harmful due to loss of regenerative capacity (108). For tissues and organs retaining regenerative competence, clarifying whether selective clearance of pathological cell populations can successfully restore physiologically normal tissue function and architecture represents an essential translational question (109). Moreover, systematically understanding CAR T cell trafficking dynamics, tissue infiltration patterns, and persistence within non-lymphoid, non-tumor tissues warrants comprehensive investigation. Local or locoregional CAR T cell delivery strategies-including intratracheal, endobronchial, or image-guided direct tissue injection approaches-may substantially enhance therapeutic efficacy while simultaneously minimizing systemic exposure and associated toxicities (110). As this promising therapeutic field continues evolving, elucidating intrinsic cellular and extrinsic microenvironmental modulators of CAR T cell therapeutic activity will prove critical for rational optimization (**Figure 4**).

## 6 Conclusion and future perspectives

*In vivo* engineered CAR T cell therapy represents a groundbreaking therapeutic approach, demonstrating promise for the treatment of pathological conditions characterized by aberrant fibroblast activation and progressive fibrosis. The therapeutic effectiveness and comprehensive safety profile of this innovative approach warrant rigorous validation through well-designed preclinical studies and carefully executed clinical trials specifically targeting fILD and IPF patient populations. The rapidly expanding therapeutic indications for CAR T cell therapies have intensified clinical demand; however, persistent manufacturing bottlenecks, supply chain constraints, and prohibitively high costs continue hindering widespread clinical adoption and equitable patient access. Additionally, therapeutic efficacy against fILD and IPF remains limited by fundamental challenges, including suboptimal CAR T cell persistence in non-lymphoid tissues, insufficient immunomodulatory capacity within fibrotic microenvironments, and unresolved safety considerations. Severe AEs-including life-threatening CRS, neurotoxicity syndromes, immune effector cell-associated hemophagocytic lymphohistiocytosis-like syndrome, and theoretical risks of secondary malignancies-currently impose significant constraints limiting broader clinical application in practice. These multifaceted challenges underscore the urgent need for the development of safer, more precisely targeted CAR molecular architectures and implementation of evidence-based toxicity mitigation strategies, including preemptive cytokine blockade and controllable CAR expression systems. Concerted efforts to systematically improve CAR T cell trafficking to fibrotic tissues, enhance long-term persistence through optimized co-stimulatory domain selection, and refine antigen specificity while maintaining acceptable safety margins prove crucial to enhancing the therapeutic sustainability and clinical translatability of this promising cellular immunotherapy modality. Addressing these formidable challenges in CAR T cell therapy applications against fILD and IPF could be substantially strengthened by articulating more concrete translational research directions and clinical development pathways. These include: (1) development of dual-antigen-targeted or Boolean logic-gated CAR architectural designs to dramatically improve target cell specificity while sparing healthy fibroblast populations; (2) implementation of inducible or transient CAR expression strategies utilizing mRNA delivery platforms or pharmacologically controllable systems to mitigate long-term toxicity risks and enable dose titration; (3) establishment of patient stratification approaches integrating advanced molecular imaging modalities (such as FAP-targeted PET imaging) and tissue/circulating molecular biomarker panels to identify optimal candidate patients most likely to benefit; and (4) rational design of synergistic combination therapeutic regimens integrating CAR T cell therapy with existing antifibrotic pharmacological agents (pirfenidone, nintedanib), targeted immunomodulatory biologics, or ECM-remodeling enzymes to achieve superior and more durable therapeutic outcomes compared to monotherapy approaches.
